# Do Sensory Stimulation Programs Have an Impact on Consciousness Recovery?

**DOI:** 10.3389/fneur.2018.00826

**Published:** 2018-10-02

**Authors:** Lijuan Cheng, Daniela Cortese, Martin M. Monti, Fuyan Wang, Francesco Riganello, Francesco Arcuri, Haibo Di, Caroline Schnakers

**Affiliations:** ^1^International Vegetative State and Consciousness Science Institute, Hangzhou Normal University, Hangzhou, China; ^2^Research in Advanced Neurorehabilitation, S. Anna Institute, Crotone, Italy; ^3^Department of Psychology, University of California, Los Angeles, Los Angeles, CA, United States; ^4^Department of Neurosurgery, David Geffen School of Medicine at UCLA, Los Angeles, CA, United States; ^5^Research Institute, Casa Colina Hospital and Centers for Healthcare, Pomona, CA, United States

**Keywords:** brain injuries, consciousness, persistent vegetative state, minimally conscious state, sensory stimulation

## Abstract

**Objectives:** Considering sensory stimulation programs (SSP) as a treatment for disorders of consciousness is still debated today. Previous studies investigating its efficacy were affected by various biases among which small sample size and spontaneous recovery. In this study, treatment-related changes were assessed using time-series design in patients with disorders of consciousness (i.e., vegetative state—VS and minimally conscious state—MCS).

**Methods:** A withdrawal design (ABAB) was used. During B phases, patients underwent a SSP (3 days a week, including auditory, visual, tactile, olfactory, and gustatory stimulation). The program was not applied during A phases. To assess behavioral changes, the Coma Recovery Scale-Revised (CRS-R) was administered by an independent rater on a weekly basis, across all phases. Each phase lasted 4 weeks. In a subset of patients, resting state functional magnetic resonance imaging (fMRI) data were collected at the end of each phase.

**Results:** Twenty nine patients (48 ± 19 years old; 15 traumatic; 21 > a year post-injury; 11 VS and 18 MCS) were included in our study. Higher CRS-R total scores (medium effect size) as well as higher arousal and oromotor subscores were observed in the B phases (treatment) as compared to A phases (no treatment), in the MCS group but not in the VS group. In the three patients who underwent fMRI analyses, a modulation of metabolic activity related to treatment was observed in middle frontal gyrus, superior temporal gyrus as well as ventro-anterior thalamic nucleus.

**Conclusion:** Our results suggest that SSP may not be sufficient to restore consciousness. SSP might nevertheless lead to improved behavioral responsiveness in MCS patients. Our results show higher CRS-R total scores when treatment is applied, and more exactly, increased arousal and oromotor functions.

## Introduction

Amantadine is till now the only treatment that has shown its efficacy in patients with severe brain injury (1). Finding new ways to treat patients recovering from disorders of consciousness is therefore one of the biggest challenge facing clinicians (2). Patients can stay during months to years in disorders of consciousness such as vegetative state (which is characterized by the presence of arousal but the absence of awareness) or minimally conscious state (which is characterized by the presence of fluctuating but reproducible signs of consciousness but an absence of reliable communication), leading to a financial and ethical conundrum for the families (3, 4). Sensory stimulation programs (SSP) have been the most studied treatment in the neurorehabilitation field (5). These programs are based on the idea that an enriched environment benefits brain plasticity and improves the recovery of injured brains (6).

Rosenzweig and coworkers who were the first to introduce “environmental enrichment” in the field of animal research four decades ago showed that the morphology and physiology of the brain can be altered by modifying the quality and intensity of environmental stimulation (7, 8). Enriched environment has been associated with changes in cortical thickness (9, 10), changes in neurons size, number and connections (11–16). Exposure to such environment has shown to be beneficial following experimental brain lesions (17–19), particularly, in terms of recovery of cognitive (e.g., learning and memory) and motor functions (20–22). Enriched environment following brain injury has also shown additional beneficial effects such as decreasing lesion size or enhancing dendritic branching (6, 23–25).

Based on animal research, the Institutes for the Achievement of Human Potential (IAHP) have introduced SSP in the field of neurorehabilitation. Despite the lack of scientific evidence in human subjects, these programs were supported on the principle that they could enhance the rehabilitative process by avoiding environmental deprivation and promoting synaptic reinnervation, thus accelerating the recovery from disorders of consciousness in severely brain injured patients (26). Numerous studies have investigated SSP in patients with disorders of consciousness [for a review see: (5, 27, 28)]. While Padilla (5) concluded that the current literature provided strong evidence that multimodal sensory stimulation improves arousal and enhances clinical outcomes for patients in a coma or persistent vegetative state, both Meyer (28) and Cossu (27) reported that there was conflicting evidence regarding the clinical relevance and the benefit of sensory stimulation in patients recovering from coma. Most studies are, indeed, affected by various methodological biases such as, among others, poor description of the disorders of consciousness, poor validity, and/or sensitivity of the outcome measure, small sample size as well as spontaneous recovery. Indeed, these studies were mostly performed in the acute stage, a period during which spontaneous recovery has the highest probability to occur. Interestingly, several recent studies investigated whether the improvements observed after SSP exceeded spontaneous recovery using a time-series design. However, they all included a small number of patients (*n* < 15) (29–31). Finally, neuroimaging data was collected in a subset of patients. Only one study recently investigated the changes in brain activity related to treatment. Pape and coworkers examined the effects of a unimodal stimulation program in 15 patients using familiar auditory stimulation and found higher activation in the language network in the treated group as compared to the control group, suggesting that coupling behavioral measures with neuroimaging may help to understand what impact sensory stimulation has on the recovering brain (32).

Therefore, the aim of this study was to assess the impact of SSP on the recovery of consciousness (as measured by the Coma Recovery Scale-Revised) and to determine treatment-related changes using a time-series design in a group of patients with disorders of consciousness (i.e., VS and MCS).

## Methods

### Inclusion/Exclusion criteria

Severely brain injured patients diagnosed as being in a vegetative state (VS) (3) or in a minimally conscious state (MCS) (4) were recruited from the Rehabilitation Center for Brain Damage of Wujing Hospital (Hangzhou, China) and the Research in Advanced Neurorehabilitation of S. Anna Institute (Crotone, Italy). Patients were only followed during their stay in the inpatient rehabilitation unit. Patients were included in the study if they (a) were at least 18 years old, (b) were at least a month post-injury, and (c) presented periods of spontaneous eye opening. Traumatic and non-traumatic etiologies were included in this study. Patients were excluded if they had (a) a documented history of prior brain injury, (b) premorbid history of uncorrected visual or hearing impairments, (c) premorbid history of developmental, psychiatric, or neurologic illness resulting in documented functional disability up to the time of the injury, (d) acute illness, (e) emerged from MCS during the first A phase as assessed by the Coma Recovery Scale—Revised (33), and (f) medical complications during the study. Information regarding patients' comorbidities and education were not collected. This study was carried out in accordance with the recommendations of the ethics committee of the Hangzhou Normal University (Hangzhou, China) and the S. Anna Institute (Crotone, Italy). The study was approved by the ethics committee of each participating center. Written informed consent was obtained from the patients' legal surrogate in accordance with the Declaration of Helsinki.

### Behavioral data acquisition and analyses

#### Procedure

Time series design was chosen to address previous criticisms on spontaneous recovery (34). Indeed, one advantage of this design is to compare baselines to treatment and, therefore, to measure how the presence/absence of the target treatment modulates the outcome measure within each participant. An ABAB withdrawal design (where A = baseline and B = treatment) was preferred to an AB or ABA design since it provided an opportunity to repeatedly collect data on the relationship between the treatment and the outcomes of interest. Each phase of this ABAB design lasted 4 weeks, as previously used (27, 29). During A phases, no SSP was administered, the patients only received until 3 h a day for 5 days a week of comprehensive rehabilitation including nursing care as well as physical therapy, respiratory therapy and speech therapy. During B phases, a SSP (described below) was also administered 3 times a week (i.e., Monday, Wednesday, and Friday; twice a day), as agreed with the medical staff.

The Coma Recovery Scale—Revised (CRS-R) (33) was chosen as our outcome measure and was administered once weekly (i.e., Saturday) for the full length of the study (i.e., across all phases) to assess changes in behavioral responsiveness. The Chinese and Italian translations of the scale were used in this study (35, 36). The CRS-R was designed to differentiate VS from MCS patients and is recommended by the American Congress of Rehabilitation Medicine to assess patients with disorders of consciousness (37). It consists of 23 hierarchically-arranged items divided into six subscales assessing auditory, visual, motor, oromotor, communication, and arousal functions. The rater performing the CRS-R assessments was not involved in the administration of the SSP and was not aware of the study design (i.e., ABAB). In each center, the same rater assessed the patients every week.

#### SSP

The administration of SSP corresponds to the B phases of our procedure. Based on the literature, we opted for a multi-sensory stimulation program including auditory, visual, tactile, olfactory, and gustatory stimuli (5, 28). Familiar stimulations were used since it has been shown that there is a higher probability to observe an improved behavioral response when emotional stimuli are presented (5, 38). Each stimulation was administered three times, on the patient's right and left side alternatively (inter-stimulus interval of 20 s). The order in which sensory stimulations were applied was randomized for each session. The program lasted around 20 min per session.

The program included the following stimulation: (a) Visual stimulation. A picture of the family member with whom the patient had the closest relationship before the injury was presented to the patient. If not possible to obtain, a picture with a high positive valence (valence of 8 according to the International Affective Picture System) was used (39). The picture was slowly moved 45 degrees to the right and left of the vertical midline and 45 degrees above and below the horizontal midline. (b) Auditory stimulation. The patient's favorite music before the injury was chosen. If not possible to obtain, classical music was used. (c) Tactile stimulation. Fingertips were used to apply firm pressure down the patient's arm, from the shoulder to the wrist. Areas with fractures as well as skin or muscular lesions were not stimulated. (d) Olfactory stimulation. The smell the patient preferred before the injury (or, by default, vanilla concentrate) was presented underneath the patient's nose. In case of tracheotomy, the entrance of the cannula was covered. (e) Gustatory stimulation. The flavor the patient preferred before the injury (or, by default, vanilla concentrate) was chosen. A stick soaked of this flavor was introduced into the patient's mouth.

Several recommendations had to be followed such as: applying the treatment while the patients were in a wakeful state with eyes open in a setting with minimal ambient noise and respecting a 30 min rest before each session (i.e., absence of nursing care).

#### Statistical analyses

A mixed-design ANCOVA was performed on the CRS-R total scores with phase (ABAB) and week (1-2-3-4) as within-subject factors, diagnosis (VS vs. MCS) and etiology (traumatic vs. non-traumatic) as between-subjects factors, and time since injury as a covariate. The effect size was estimated, for each significant result, using a partial-η^2^ statistic (small: η_*p*_^2^ ≥ 0.01; medium: η_*p*_^2^ ≥ 0.06; large: η_*p*_^2^ ≥ 0.14) (40). Planned comparisons were intended to be used to compare CRS-R total scores during B phases (treatment) vs. A phases (no treatment) for both VS and MCS groups but also within each group, separately. Wilcoxon tests were performed to compare CRS-R mean subscores during A phases and B phases for both VS and MCS groups but also within each group, separately.

### fMRI data acquisition and analyses

Neuroimaging data were acquired at one of the two centers which had Magnetic Resonance Imaging (MRI), the Rehabilitation Center for Brain Damage of Wujing Hospital (Hangzhou, China). Using a 1.5 Tesla Siemens Magnetom Essenza MRI system (Siemens AG, Munich, Germany). Resting state functional MRI (fMRI) data were collected in a subset of patients at the end of each phase (on the fourth week) to assess the effects of the treatment on brain activity. Inclusion criteria were: stability of vital parameters and absence of contra-indications for entering the MRI environment. The preprocessed data was used to calculate, on a single-subject basis, the Amplitude of Low Frequency Fluctuations (ALFF) across the whole brain (band frequency of interest: 0.01–0.1 Hz). Results were Z-scored across the full brain. Group data was analyzed using a repeated measures ABAB design, to assess the effect of phase on the ALFF maps. For more information regarding the fMRI acquisition and analyses, see Supplementary Material.

## Results

### Participants

Twenty nine patients (48 ± 19 years old; 19 men; age range: 20–79 years) were included in this study. The etiology of brain injury was traumatic (*n* = 15), anoxic (*n* = 5), ischemic stroke (*n* = 5), hemorrhagic (*n* = 3), or metabolic (*n* = 1). The time since injury was more than a year for 21 patients (1.04–10.7 years) and less than a year for eight patients (41–348 days). According to CRS-R scores (Table 1), 11 patients presented a stable diagnosis of VS and 18 patients presented a stable diagnosis of MCS during the first A phase. Eighteen patients were recruited at the Rehabilitation Center for Brain Damage of Wujing Hospital (Hangzhou, China) and 11 at Research in Advanced Neurorehabilitation, S. Anna Institute (Crotone, Italy). To test differences in patients' population among both centers, *T*-tests and Chi-squares were used to compare variables that are known to impact patients' outcome (2): time since injury, etiology (i.e., traumatic vs. non-traumatic) and diagnosis (i.e., VS vs. MCS). We did not find any difference between centers (*t*_(27)_ = 1.53, *p* = 0.14; $\chi_{\left(1\right)}^{2}$ = 0.96, *p* = 0.33; and, $\chi_{\left(1\right)}^{2}$ = 1.65, *p* = 0.2, respectively). The medications most frequently administered included; antispastics, anticonvulsants, anti-acid, laxatives, analgesics, mucolytics, vitamins, and supplements. None of our patients received Amantadine (or Zolpidem), which could have an impact on the patient's consciousness recovery (1, 2).

**Table 1:** Demographic data for minimally conscious (MCS) and vegetative (VS) patients.

**Patient**	**Etiology**	**TSI**	**CRS-R**	**AF**	**VF**	**MF**	**OF**	**C**	**Ar**
MCS 1	Traumatic	4.8*y*	13	2	3	5	1	0	2
MCS 2	Traumatic	3.2*y*	10	2	3	2	1	0	2
MCS 3	Traumatic	4.48*y*	10	0	3	5	0	0	2
MCS 4	Stroke	2.87*y*	13	2	3	4	2	0	2
MCS 5	Stroke	2.93*y*	10	2	3	2	1	0	2
MCS 6	Traumatic	2.93*y*	9	2	3	2	1	0	1
MCS 7	Stroke	2.91*y*	15	4	5	2	1	1	2
MCS 8	Anoxic	1.04*y*	19	4	5	5	2	1	2
MCS 9	Traumatic	2.4*y*	10	2	3	2	1	0	2
MCS 10	Traumatic	5.52*y*	13	2	3	5	1	0	2
MCS 11	Hemorrhage	10.07*m*	9	1	3	2	1	0	2
MCS 12	Stroke	3.53*m*	13	3	1	5	2	0	2
MCS 13	Traumatic	1.27*y*	9	2	1	2	2	0	2
MCS 14	Traumatic	5.47*m*	8	2	2	2	0	0	2
MCS 15	Traumatic	11.6*m*	14	3	4	4	1	0	2
MCS 16	Traumatic	6.9*m*	5	0	0	4	1	0	0
MCS 17	Traumatic	2.19*y*	11	2	3	2	2	0	2
MCS 18	Anoxic	2.42*y*	12	1	3	5	1	0	2
VS 1	Traumatic	3.95*y*	8	1	1	2	2	0	2
VS 2	Traumatic	1.45*y*	7	1	1	2	1	0	2
VS 3	Hemorrhage	1.09*y*	6	2	0	2	1	0	1
VS 4	Hemorrhage	1.81*y*	7	2	0	2	1	0	2
VS 5	Traumatic	7.23*m*	6	0	0	2	2	0	2
VS 6	Metabolic	5.36*y*	7	2	0	2	1	0	2
VS 7	Anoxic	3.77*m*	7	1	1	2	2	0	1
VS 8	Stroke	1.28*y*	7	1	0	2	2	0	2
VS 9	Traumatic	1.33*y*	7	1	1	2	1	0	2
VS 10	Anoxic	1.37*m*	8	2	1	2	1	0	2
VS 11	Anoxic	10.7*y*	7	1	0	2	2	0	2

*TSI, Time Since Injury (y = years/m = months); CRS-R, total scores for the Coma Recovery Scale-Revised (AF, Auditory Function; VF, Visual Function; MF, Motor Function; OF, Oromotor Function; C, communication; Ar, Arousal). The highest CRS-R total scores (and its subscores) on the first A phase (baseline) are mentioned*.

### Behavioral results

**Figure 1:**
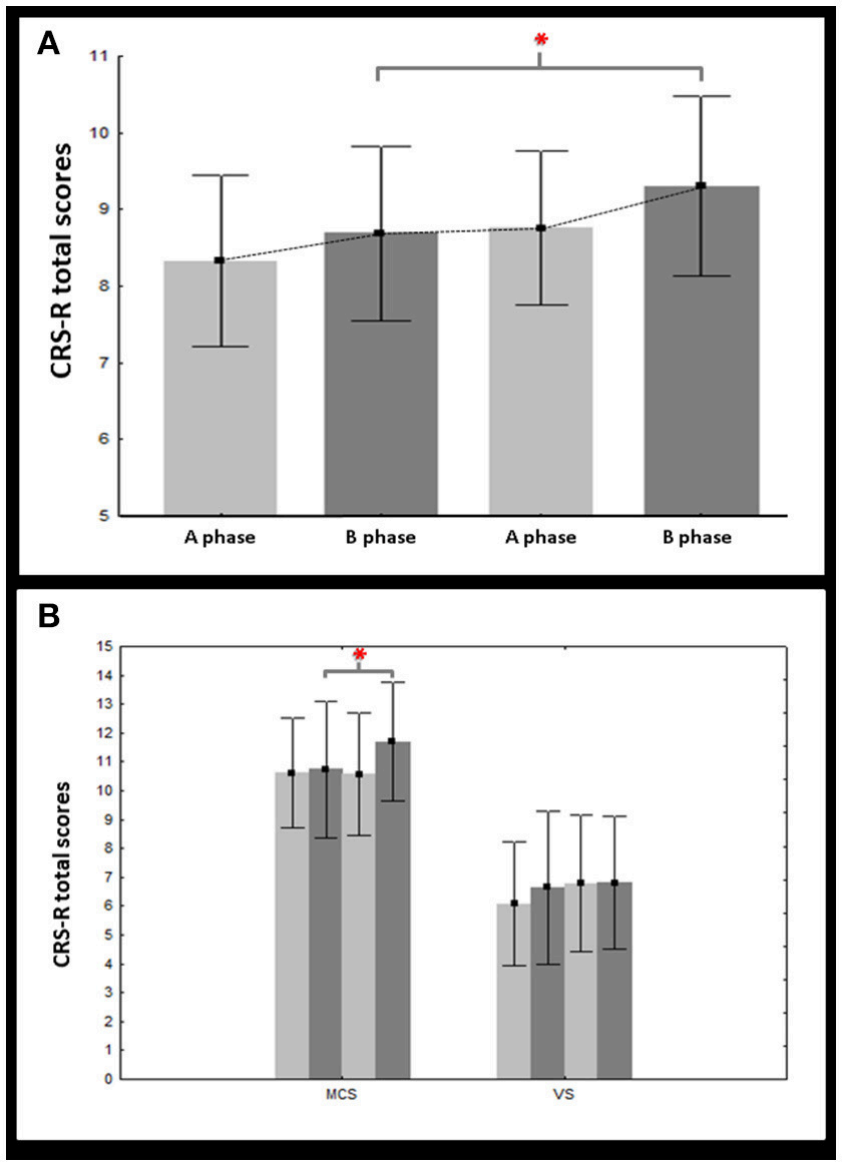
Changes in CRS-R total scores. This figure illustrates the mean (bars = 95% confidence intervals) of the CRS-R total scores on treatment (dark gray) vs. off treatment (light gray) for both vegetative (VS) and minimally conscious (MCS) groups **(A)** but also within each group, separately **(B)**. Asterisks indicate significant results (*p* < 0.05).

Using a mixed-design ANCOVA, a main effect of phase (ABAB) [*F*_(3)_ = 3.17, *p* = 0.03] was found. The effect size was found to be medium (η_*p*_^2^ = 0.12). We did not find any interaction with the time since injury [*F*_(3)_ = 0.65, *p* = 0.58], the etiology (i.e., traumatic vs. non traumatic) [*F*_(3)_ = 0.36, *p* = 0.78], or the diagnosis [*F*_(3)_ = 1.35, *p* = 0.26]. We have to note that we also found a main effect of the diagnosis [*F*_(1)_ = 39.78, *p* < 0.001], which is not surprising since this variable (particularly, being conscious/MCS) is known to impact patients' general outcome (41) (Supplementary Material and Figure 1). Using planned comparisons, we found a significant difference [*F*_(1)_ = 6.98, *p* = 0.01] between B phases (treatment) and A phases (no treatment); CRS-R total scores being higher during treatment. However, when considering the diagnosis, CRS-R total scores were found to be higher during treatment in MCS patients [*F*_(1)_ = 7.18, *p* = 0.01] but not in VS patients [*F*_(1)_ = 1.28, *p* = 0.27] (Figure 1).

Regarding subscores, higher scores during treatment (B phases vs. A phases) were only found for the oromotor subscale (*T* = 2.73, *p* = 0.006) and the arousal subscale (*T* = 2.8, *p* = 0.005). Such difference was confirmed in MCS patients (*T* = 2.07, *p* = 0.04 and *T* = 2.22, *p* = 0.03, respectively) but not in VS patients (Table 2).

**Table 2:** Results for the Wilcoxon tests performed to compare CRS-R subscores (average ± standard deviation) during A phases and B phases for both VS and MCS groups but also within each group, separately.

**MCS/VS**	**A phases**	**B phases**	***p***
Auditory	1.33 ± 0.71	1.35 ± 0.80	0.95
Visual	1.82 ± 1.39	1.84 ± 1.35	0.64
Motor	2.51 ± 1.11	2.56 ± 1.19	0.57
Oromotor	1.20 ± 0.38	1.34 ± 0.41	0.006*
Communication	0.04 ± 0.16	0.08 ± 0.32	0.18
Arousal	1.74 ± 0.26	1.83 ± 0.19	0.005*
**MCS**			
Auditory	1.49 ± 0.83	1.60 ± 0.88	0.30
Visual	2.63 ± 1.12	2.60 ± 1.08	0.97
Motor	2.94 ± 1.23	3.03 ± 1.28	0.36
Oromotor	1.18 ± 0.45	1.33 ± 0.47	0.04*
Communication	0.07 ± 0.20	0.13 ± 0.40	0.18
Arousal	1.82 ± 0.26	1.91 ± 0.17	0.03*
**VS**			
Auditory	1.07 ± 0.36	0.95 ± 0.43	0.08
Visual	0.49 ± 0.37	0.60 ± 0.62	0.39
Motor	1.82 ± 0.17	1.78 ± 0.35	0.68
Oromotor	1.21 ± 0.24	1.36 ± 0.33	0.07
Communication	0 ± 0	0 ± 0	1
Arousal	1.62 ± 0.21	1.7 ± 0.14	0.08

*Significant results are indicated by an asterisk (p < 0.05)*.

### fMRI results

**Figure 2:**
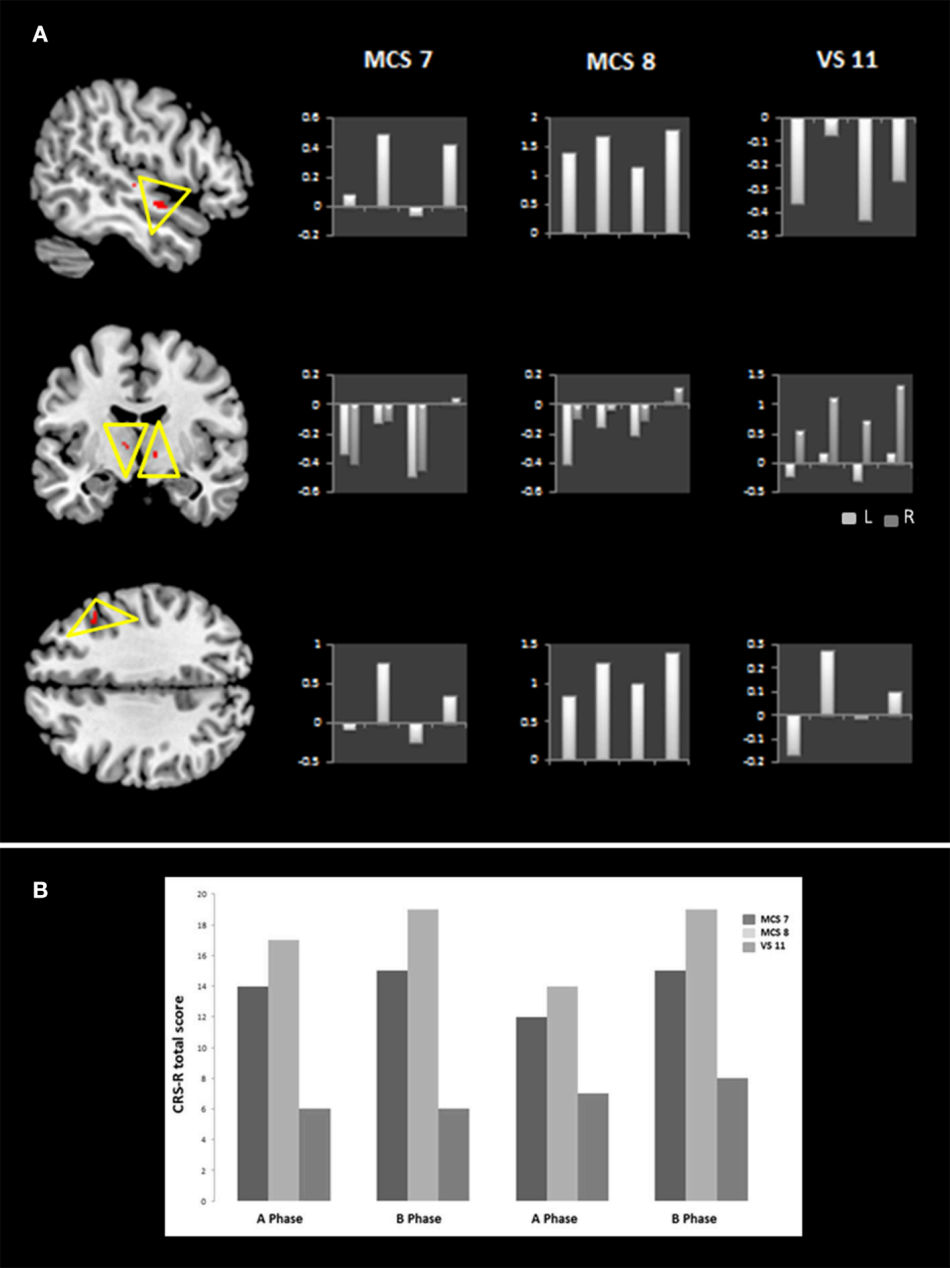
Brain areas with treatment-related metabolic changes. The left side of **(A)** illustrates, at the group level, areas with treatment-related metabolic changes which include the right middle frontal gyrus, the right superior temporal gyrus as well as the bilateral ventro-anterior thalamic nucleus (L = left, R = right) (*p* < 0.005 voxel-wise uncorrected). On the right side of **(A)**, z-scores for each activated area are also reported at each phase (ABAB) for patients MCS 7, MCS 8, and VS 11. **(B)** Shows the CRS-R total scores on the last week of each phase (ABAB) for patients MCS 7, MCS 8, and VS 11.

fMRI scans were performed across each phase on seven patients. Patients who exhibited motion greater than one voxel (i.e., 3 mm) were excluded from the analysis. Therefore, data of only three patients (i.e., MCS 7, MCS 8, and VS 11) were considered for analyses. Because of the small number of patients, group analyses were performed using a fixed-effects model (42), and significance was assessed using a non-parametric permutation test (available in FSL) at a significance level of *p* < 0.005 uncorrected. Regions exhibiting significant activations were identified using the MNI structural atlas, and further specified with the Harvard-Oxford atlas and the ICBM Deep Nuclei Probabilistic atlas (43, 44). The group ALFF analyses revealed higher activation during treatment in the right middle frontal gyrus (*t* = 1.71, *p* = 0.001; peak voxel: x = 21, y = 70, z = 54) and right superior temporal gyrus (*t* = 1.88, *p* = 0.001; peak voxel: x = 20, y = 62, z = 31) as well as the bilateral ventro-anterior thalamic nucleus (*t* = 1.26/1.23, *p* = 0.002/0.003; peak voxels: x = 49/40, y = 59/59, z = 38/35, for the left and right hemispheres, respectively) (Figure 2).

## Discussion

The aim of this study was to assess the impact of SSP on the recovery of consciousness and to determine treatment-related changes using a time-series design in patients with disorders of consciousness (i.e., VS and MCS). Our results suggest that SSP may not be sufficient to restore consciousness. However, SSP might lead to improved behavioral responsiveness in MCS patients. Our results show higher CRS-R total scores when treatment is applied with increased arousal and oromotor function but no changes for the other subscales (i.e., visual, motor, or communication).

Our results showed higher CRS-R total scores when treatment was applied (B phases) as compared to when treatment was not applied (A phases) (with a medium effect size). The time since injury or the etiology did not seem to have an impact on our dataset. However, patients who were diagnosed as being in a MCS obtained higher CRS-R total scores during treatment than off treatment while patients who were diagnosed as being in a VS did not show such changes. We should however, nuance our findings since we did not find an interaction between CRS-R changes through each phases and diagnosis (VS/MCS) which indicates that the significant changes that we found for our planned comparison (when compiling both A phases versus both B phases) are in fact smaller when each phase is considered separately. Higher CRS-R subscores were also found during treatment for the oromotor and arousal subscales but not for the other subscales (i.e., visual, motor, or communication). These findings were mainly present in MCS patients (i.e., higher oromotor and arousal subscores during treatment) but not in VS patients. We have to mention that even though we found significant results in two subscales of the CRS-R, we could not show, because of statistical limitation, that these changes were significantly higher as compared to the other subscales. Therefore, specific treatment related improvements in arousal and verbal functions should be further investigated and confirmed in the future.

Previous studies using time series and performed in smaller samples (*n* < 15) have reported a modulation of behavioral responses related to treatment based on standardized scales assessing the level of consciousness (29, 30). Using emotionally relevant multi-modal stimulation, Di Stefano and coworkers have found an increased responsiveness in terms of the number of behaviors but also in terms of complexity, based on the Wessex Head Injury Matrix (45). Additionally, even though it did not reach significance, MCS tend to show more behavioral responsiveness than VS patients similarly to our findings. The authors also suggested that the use of emotional stimuli (as used in this study) might have optimized arousal and facilitates behavioral responsiveness (30). Improved arousal has also been shown in studies using a controlled design based on the Glasgow Coma Scale (28, 46, 47). Finally, recent studies have shown that SSP using emotional stimuli have a higher likelihood to lead to increased responsiveness while studies using neuroimaging showed higher metabolic brain activity in response to self-relevant stimuli (5, 38, 48).

The behavioral changes observed (increased arousal and oromotor function) also seem to be in line with our neuroimaging data. Indeed, treatment-related metabolic changes were observed in the superior temporal gyrus, the middle frontal gyrus, and the ventral anterior thalamic nucleus. The superior temporal and middle prefrontal gyri are typically recruited by a number of cognitive processes including language (49) while the ventral anterior thalamic nucleus is known to be a major source of projection to the premotor sections of the frontal cortex, and is involved in motor planning and speech (50). The thalamus also plays a key role in arousal and consciousness. According to the mesocircuit theory, projections from thalamus to associative cortical areas (including temporal and frontal) are crucial for sustaining organized behaviors and integrating information across different regions of cortex (51). Moreover, the ventral anterior nucleus receives neuronal inputs from the basal ganglia which seems to serve a critical role in the maintenance of behavioral and electrocortical arousal, as well as wakefulness (52–54). Finally, Pape and coworkers found higher activation in the superior temporal and prefrontal gyri in the treated group as compared to the control group, following an unimodal (familiar auditory) stimulation. Anecdotally, the authors also observed higher arousal and more vocalizations in the treated group (32). Besides the neuroimaging modulation observed, the behavioral results obtained in the patients who underwent fMRI, particularly, MCS7 and MCS8 (who were chronic non-traumatic patients; respectively, 2.91 and 1.04 y post injury), also seem to show fluctuations according to the presence/absence of treatment. VS 11 (who was chronic non-traumatic patient; 10.7 y post injury) did not show such fluctuations (but a constant increase which is difficult to interpret as related to our treatment). This observation is parallel to what we found at the group level since changes in CRS-R scores were mainly observed in the MCS group. Nevertheless, we have to nuance this interpretation since VS 11 had a significantly longer time post injury (10 y), which might also explain why we don't see changes related to treatment. We also have to stress that our neuroimaging findings were based on an extremely small subsample (*n* = 3), which makes it difficult to formulate firm conclusions. Nonetheless, except Pape et al. (32), no studies have reported neuroimaging findings. Such findings are important since they allow us to start better understanding the mechanisms of action of a particular treatment, here, the SSP. We nevertheless do realize that the generalization of these results is quite limited and that these data are very preliminary.

Our study aimed to address various methodological biases existing in previous studies such as poor description of disorders of consciousness, poor validity, and/or sensitivity of the outcome measure, small sample size as well as spontaneous recovery. Patients recruited in this study were assessed and diagnosed either in a VS or in a MCS based on the CRS-R. The CRS-R is currently the most validated and sensitive scale available to perform behavioral assessment in patients with severe brain injury and to stratify with high accuracy the level of consciousness (37). On the other hand, time series withdrawal design was also chosen not only to address the sample size but also the spontaneous recovery issue. Indeed, withdrawal designs (here, ABAB) provided a high degree of experimental control while being relatively straightforward to plan and implement. Such design allows to repeatedly compare baseline to treatment in order to measure the outcome with and without the intervention, and therefore offers a better control for the impact of natural recovery. Another advantage is that, as compared to controlled designs, within-subject measures requests no matching processes and a smaller sample size (since the sample is not divided between an intervention and a control group). Our study is the first one to include a large sample (*n* = 29) using time-series design, confirming previous preliminary findings (29–31). Only one recent study using a controlled design included 30 patients per group. Indeed, Salmani et al. (48) evaluated the effects of SSP including emotional stimulation on the level of consciousness and showed higher GCS and CRS-R total scores in the end of the intervention in patients receiving emotional SSP as compared to neutral SSP, suggesting that family-centered affective stimulation is more effective in improving the level of consciousness.

This study has several limitations. First, even though the time since injury did not seem to influence the behavioral changes observed during treatment, the majority of our patients were chronic (21 of 29 patients were more than a year post injury), decreasing our chances to see spontaneous recovery but most likely also reducing our chances to see consciousness improvement. Also, the size of VS (*n* = 11) and MCS (*n* = 18) groups did not allow us to explore further the difference of outcome observed. Our neuroimaging data was collected in a very small sample limiting the interpretation of our findings. A double-blinded design was not used in this study. The CRS-R rater was blinded regarding the ABAB design but knew that the study was about applying treatment to patients with disorders of consciousness. The therapist applying the SSP and the patients' family were not blinded. The aim of the study was not to determine the time, the frequency, the duration and type of program (multi-modal, unimodal, or sensory regulation) (47) requested to optimize the recovery of patients with severe brain injury. One could argue that the effects observed might be due to changes in therapy independent from our treatment. Nevertheless, changes might likely have happened at random in our sample (*n* = 29); i.e., it is most likely that not all our patients stopped or started a therapy at the same time but rather stopped or started a therapy at different time through the study. The withdrawal design we used allowed us to look at behavioral changes that are time locked to treatment as opposed to random changes in treatment. Besides, the statistics we used (mixed design ANCOVA) also controls for such bias and ensures that the effects observed represent a global tendency of the group (that is time locked to treatment). Our data cannot speak on whether the presently applied SSP also leads to lasting changes in level of consciousness and therefore really leads to lasting rehabilitation benefits (in contrast to short-lived changes in responsiveness that might vanish as soon as the SSP is discontinued). Future studies will have to include long term follow-up in order to answer this important issue. Finally, since a controlled design was not used, one cannot clearly determine, in this study, whether improvements are due to specific aspects of our SSP (such as emotional stimulation) or non-specific aspects (such as more time devoted to patients, or non-specific arousal effect).

In conclusion, our study showed a modulation of behavioral responses in a larger sample using time series design. Our results suggest that, even if it may not be sufficient to restore consciousness, SSP might lead to improved behavioral responsiveness in MCS patients. Combined with other validated therapeutics (such as Amantadine) (1), SSP might optimize patients' recovery. Further investigation is nevertheless warranted to test this hypothesis.

## Author contributions

CS and HD: conception, design, and supervision of the work; LC, FW, DC, FR, and FA: acquisition and analysis of behavioral data; FW and MM: acquisition and analysis of fMRI data; CS and HD: interpretation of data for the work; LC, DC, and CS: drafting the work; MM, FW, FR, FA, and HD revising critically for important intellectual content.

### Conflict of interest statement

The authors declare that the research was conducted in the absence of any commercial or financial relationships that could be construed as a potential conflict of interest.
