# Supplementary material for: Do Sensory Stimulation Programs Have an Impact on Consciousness Recovery?
Source: Front Neurol. 2018 Oct 2;9:826. doi: 10.3389/fneur.2018.00826 (PMC6176776; doi:10.3389/fneur.2018.00826)
Supplement: Supplementary file 1 [file Table_1.doc]

**Supplementary material:**

1. Results for the mixed-design ANCOVA performed on the CRS-R total scores with phase (ABAB) and week (1-2-3-4) as within-subject factors, diagnosis (“Diagn”; VS vs. MCS) and etiology (“Etio”; traumatic vs. non-traumatic) as between-subjects factors, and time since injury (“TSI”) as a covariate. Significant results are indicated by an asterisk (p<.05).

|  | d | F | p |
| --- | --- | --- | --- |
| {3}TSI | 1.00 | 0.01 | 0.92 |
| {1}Etio | 1.00 | 1.40 | 0.25 |
| {2}Diagn | 1.00 | 39.78 | 0.00* |
| Etio*Diagn | 1.00 | 4.20 | 0.05 |
|  |  |  |  |
| {4}Phase | 3.00 | 3.17 | 0.03* |
| Phase*TSI | 3.00 | 0.65 | 0.58 |
| Phase*Etio | 3.00 | 0.36 | 0.78 |
| Phase*Diagn | 3.00 | 1.35 | 0.26 |
| Phase*Etio*Diagn | 3.00 | 1.16 | 0.33 |
|  |  |  |  |
| {5}Week | 3.00 | 0.34 | 0.80 |
| Week*TSI | 3.00 | 0.30 | 0.82 |
| Week *Etio | 3.00 | 0.26 | 0.85 |
| Week *Diagn | 3.00 | 1.01 | 0.39 |
| Week *Etio*Diagn | 3.00 | 0.07 | 0.98 |
|  |  |  |  |
| Phase * Week | 9.00 | 0.51 | 0.86 |
| Phase * Week *TSI | 9.00 | 0.31 | 0.97 |
| Phase * Week *Etio | 9.00 | 0.59 | 0.80 |
| Phase * Week *Diagn | 9.00 | 0.61 | 0.79 |
| 4*5*1*2 | 9.00 | 0.88 | 0.54 |

1. fMRI acquisition and analyses

T2* sensitive functional data were collected using a gradient echo planar imaging (GRE) sequence in 22 ascending slices (TR = 3000 ms, TE=60 ms, flip angle 90°, FOV = 240 x240 mm, voxel size 3.75x3.75x6, and matrix size 64x64) for a total of 200 volumes (i.e., 10 minutes). Structural T1-weighted images were acquired using a spin echo (SE) sequence (TR = 500 ms, TE = 14 ms, FOV = 240x240 mm, slice thickness = 5 mm, skip = 1 mm, matrix size 256x256, voxel size 0.9375x0.9375x6).

The initial 4 TRs of each functional dataset were removed to allow for stabilization of the blood oxygen level dependent (BOLD) signal. Following, data underwent slice-time correction, rigid-body adjustment for intra-run motion, brain extraction, 8 mm FWHM smoothing, band-pass filtering (0.008 <Hz< 0.1), grand mean scaling (to a value of 1000) and removal of linear and quadratic trends. Nuisance signals, including six motion parameters (Power et al., 2014), white matter and CSF associated time-courses (but not the global mean signal) were partialled-out using a linear regression. The residuals of the regression were then co-registered to the MNI template available in FSL (FMRIB's Software Library, www.fmrib.ox.ac.uk/fsl) using a 12 degrees of freedom affine transformation.
